# Crosstalk between AMPK activation and angiotensin II-induced hypertrophy in cardiomyocytes: the role of mitochondria

**DOI:** 10.1111/jcmm.12220

**Published:** 2014-01-20

**Authors:** Jessica Soto Hernández, Giselle Barreto-Torres, Andrey V Kuznetsov, Zaza Khuchua, Sabzali Javadov

**Affiliations:** a Department of Physiology, School of Medicine, University of Puerto RicoSan Juan, PR, USA; b Department of Cardiac Surgery, Cardiac Surgery Research Laboratory, Innsbruck Medical UniversityInnsbruck, Austria; c Cincinnati Children's Research Foundation, University of CincinnatiCincinnati, OH, USA

**Keywords:** AMP kinase, metformin, mitochondria, angiotensin II receptors, hypertrophy

## Abstract

AMP-kinase (AMPK) activation reduces cardiac hypertrophy, although underlying molecular mechanisms remain unclear. In this study, we elucidated the anti-hypertrophic action of metformin, specifically, the role of the AMPK/eNOS/p53 pathway. H9c2 rat cardiomyocytes were treated with angiotensin II (AngII) for 24 hrs in the presence or absence of metformin (AMPK agonist), losartan [AngII type 1 receptor (AT1R) blocker], Nω-nitro-L-arginine methyl ester (L-NAME, pan-NOS inhibitor), splitomicin (SIRT1 inhibitor) or pifithrin-α (p53 inhibitor). Results showed that treatment with metformin significantly attenuated AngII-induced cell hypertrophy and death. Metformin attenuated AngII-induced activation (cleavage) of caspase 3, Bcl-2 down-regulation and p53 up-regulation. It also reduced AngII-induced AT1R up-regulation by 30% (*P* < 0.05) and enhanced AMPK phosphorylation by 99% (*P* < 0.01) and P-eNOS levels by 3.3-fold (*P* < 0.01). Likewise, losartan reduced AT1R up-regulation and enhanced AMPK phosphorylation by 54% (*P* < 0.05). The AMPK inhibitor, compound C, prevented AT1R down-regulation, indicating that metformin mediated its effects *via* AMPK activation. Beneficial effects of metformin and losartan converged on mitochondria that demonstrated high membrane potential (Δψ_m_) and low permeability transition pore opening. Thus, this study demonstrates that the anti-hypertrophic effects of metformin are associated with AMPK-induced AT1R down-regulation and prevention of mitochondrial dysfunction through the SIRT1/eNOS/p53 pathway.

## Introduction

AMP-kinase (AMPK), an important metabolic sensor, regulates energy metabolism in organs and tissues including the liver, adipose tissue, and skeletal and cardiac muscles 1,2. In response to various intra- and extracellular stimuli, it enhances fatty acid uptake, and oxidative metabolism, glucose transport, glycolysis and mitochondrial biogenesis. Metformin, a biguanide drug with pleiotropic actions widely used for the treatment of patients with type 2 diabetes 3,4, is a powerful activator of AMPK. Apart from its anti-hyperglycaemic effects, metformin significantly improves cardiac function in patients and various animal models of heart failure 5, although underlying mechanisms of action remain unclear. For example, in mice with heart failure induced by left coronary artery occlusion and subsequent reperfusion, metformin improved LV function and attenuated cardiac hypertrophy 6. Similarly, metformin attenuated oxidative stress-induced cardiomyocyte apoptosis and prevented the progression of heart failure in dogs 7. However, metformin did not exert anti-hypertrophic effects on mice lacking functional AMPK or eNOS, which suggests that its effects are mediated *via* activation of the AMPK/eNOS pathway 6.

Cardiomyocyte hypertrophy, a major consequence of pressure or volume overload, plays a central role in the progression of heart failure. Various paracrine and autocrine factors are involved in pathogenesis and regulation of cardiomyocyte hypertrophy, including angiotensin II (AngII), a key player of the renin–angiotensin system that induces cell hypertrophy, differentiation and apoptosis through activation of various intracellular signalling molecules including Gq protein, calcineurin, mitogen-activated protein kinases (MAPK) and several transcription factors 8. AngII type 1 (AT1R) and type 2 (AT2R) G protein–coupled receptors have been shown to mediate the effects of circulating and local (intracellular) AngII 9,10. AT1R mediates pro-hypertrophic effects of AngII, but AT2R, in contrast, attenuates AT1R activation–induced cell growth. Recent studies demonstrated that pro-hypertrophic effects of AngII can be mediated through mitochondria and induce cell death 11. Although the exact underlying mechanisms remain unclear, AngII-induced depolarization of the mitochondrial membrane and increased production of mitochondrial reactive oxygen species (ROS) are associated with cardiomyocyte autophagy and hypertrophy 12.

Metformin, a promising pharmacological agent, may be used for the treatment of heart failure. Most previous studies were conducted *in vivo* on intact hearts. However, the heart contains many non-cardiomyocyte cell types, including fibroblasts, vascular endothelial cells, smooth muscle cells and immune cells, with cardiomyocytes accounting for only 30–40% of total heart cells. Accordingly, new studies using cultured cardiomyocytes are required to establish whether metformin exerts a direct anti-hypertrophic effect on these cells.

In this study, we elucidated the role of AMPK in AngII-induced hypertrophy in cultured H9c2 cardiomyocytes, providing additional evidence that AMPK activation improves mitochondrial function. The results demonstrate that metformin exerted anti-hypertrophic effects on cardiomyocytes and prevented AngII-induced cell death. We observed a negative reciprocal interaction between AMPK activation and AT1R levels: metformin activated AMPK, annulling AngII-induced up-regulation of AT1R, whereas losartan (AT1R antagonist) enhanced AMPK activation in AngII-treated H9c2 cardiomyocytes. Furthermore, metformin attenuated mitochondrial dysfunction and hypertrophy through the eNOS/SIRT1/p53 pathway.

## Material and methods

### Cell culture

H9c2 embryonic rat cardiomyocytes were purchased from the American Type Culture Collection (Manassas, VA, USA) and cultured according to the manufacturer's protocol. In short, the cells were cultured in medium containing DMEM/Ham's F-12 (Invitrogen, Carlsbad, CA, USA), supplemented with 10% foetal bovine serum, 10 μg/ml transferrin, 10 μg/ml insulin, 10 ng/ml selenium, 1% penicillin and streptomycin, 2 mg/ml bovine serum albumin, 5 μg/ml linoleic acid, 3 mM pyruvic acid, 0.1 mM minimum essential medium non-essential amino acids, 10% MEM vitamin, 0.1 mM 5-bromo-2′-deoxyuridine, 100 μM L-ascorbic acid and 30 mM HEPES, pH 7.1, and maintained in 95% air and 5% CO_2_ at 37°C. Prior to all experiments, cells were serum-starved for 24 hrs. Cells with 85–90% confluence from passages 3–20 were used for experiments.

### Experimental protocol

Cells were treated with 200 nM AngII for 24 hrs in the presence or absence of 2 mM metformin (AMPK activator), 10 μM losartan (AngII type 1 receptor antagonist), 5 μM compound C (AMPK inhibitor), 300 μM Nω-nitro-L-arginine methyl ester (L-NAME, pan-NOS inhibitor), 10 μM splitomicin (SIRT1 inhibitor) or 10 μM pifithrin-α (p53 inhibitor). All drugs were purchased from Sigma-Aldrich (St. Louis, MO, USA) and added to the culture medium 45 min. before AngII administration.

### Cell viability

Cell viability was determined using the trypan blue exclusion method. The cells were cultured at a density of 5 × 10^5^ cells on 100-mm dishes and exposed to AngII (0 nM, 200 nM, 500 nM, 1 μM or 5 μM) for 24 hrs in the presence or absence of 2 mM metformin. After treatment, cells were rinsed with PBS, detached with Hyclone Trypsin (Thermo Fisher Scientific, Waltham, MA, USA) and counted using the TC20 Automated Cell Counter (Bio-Rad, Hercules, CA, USA). The per cent of viable and dead cells was calculated from a total number of counted cells.

### Cell area measurement

Plated cells (3 × 10^4^ cells per 6-cm dish) were treated in accordance with the experimental protocol. At the end of the experiment, the cells were viewed using a Leica DMIL LED inverted microscope (Wetzlar, Germany), equipped with a 3.2 MP scientific grade digital microscopy camera. Six to eight random photographs were taken from each dish, and at least, 60 individual cell size measurements were made from each group using Accu-Scope Micrometrics SE Premium software.

### SDS-PAGE and Western blotting

Following the treatments, cells were washed twice with cold PBS and lysed in buffer containing 50 mM Tris-HCl, pH 7.5, 150 mM NaCl, 10 mM tetrasodium phosphate, 40 mM β-glycerophosphate, 2 mM ethylenediaminetetraacetate (EDTA), 2 mM ethylene glycol-bis(2-aminoethylether)-*N*,*N*,*N*′,*N*′-tetraacetate (EGTA), 1% Triton X, 10% glycerol, 50 mM sodium fluoride, 0.2 mM sodium orthovanadate and protease inhibitors. The cell lysates were transferred to 1.5-ml Eppendorf tubes, homogenized and centrifuged at 10,000 × g for 5 min. at 4°C. The supernatant was transferred to a fresh tube, and protein concentration was determined using the Bio-Rad Protein Assay Reagent (Bio-Rad). Equal amounts of protein samples were resolved on SDS-PAGE and transferred onto Amersham Hybond ECL 0.45 μM nitrocellulose membranes (GE Healthcare Bio-Sciences, Piscataway, NJ, USA). The membranes were immunoblotted with actin, AMPK, P-AMPKα^Thr172^, Bcl-2, caspase 3 full length, eNOS, P-eNOS (Cell Signaling, Danvers, MA, USA), AT1R, AT2R or p53 (Santa Cruz, Dallas, TX, USA) antibodies, followed by secondary antibodies. The signals were visualized using Thermo Scientific Pierce ECL Western Blotting Detection reagents (Thermo Fisher Scientific) at VersaDoc 3000 Gel Imaging System (Bio-Rad).

### Mitochondrial membrane potential

To monitor ΔΨ_m_, cells (4 × 10^5^ cells/well) plated in a 24-well culture plate were incubated for 30 min. with the membrane potential–sensitive dye JC-1 (10 μg/ml, 5,5′,6,6′-tetraethyl-benzimidazolylcarbocyanine iodide, Molecular Probes, Eugene, OR, USA). The intensity of fluorescence was immediately measured using a Spectramax M3 microplate reader (Molecular Devices) at 527 and 590 nm for emission and 488 nm for excitation. For confocal microscopy, the cells, plated on glass-bottom dishes, were loaded with JC-1 for 30 min. at 37°C, and images were captured using a Zeiss LSM510 META (Carl Zeiss, Oberkochen, Germany) microscope.

### Mitochondrial permeability transition pore (PTP)

To quantify mitochondrial PTP opening, cardiomyocytes were loaded with 5 μM calcein-acetoxymethylester (calcein-AM, Molecular Probes). Cobalt chloride (5 mM) was added to the culture medium to quench cytosolic and nuclear calcein. Cells were visualized with an Olympus IX73 inverted fluorescence microscope (Center Valley, PA, USA), and fluorescence was captured using an Olympus DP73 high-performance Peltier cooled digital colour camera. Olympus CellSens Dimension Imaging software was used to analyse calcein fluorescence in cells.

### Complex I activity

Complex I activity was determined by measuring the decrease in the concentration of NADH at 340 nm and 30°C. Cells were resuspended in hypotonic phosphate buffer containing 5 mM MgCl_2_ and 0.55 mg/ml saponin and, then, freeze-thawed three times to give the substrate access to the inner mitochondrial membrane. The assay was performed in phosphate buffer containing 1 mM KCN, 5 mM MgCl_2_, 2.5 mg/ml BSA, 2 μM antimycin, 100 μM decylubiquinone and 100 μM NADH, pH 7.4. Specific activities were determined by calculating the slope of the reaction in the linear range and normalizing per mg of protein.

### Total ROS levels

Cells were trypsinized with a 0.25% trypsin–EDTA solution (Thermo Scientific HyClone, Logan, UT, USA) and centrifuged at 500 × *g* for 5 min. at room temperature. The pellet was resuspended in culture medium, and cells were incubated with 20 μM 2′7 dichlorofluorescein diacetate (DCF-DA; Alexis Biochemicals) for 30 min. at 37**°**C. The cells were again centrifuged at 500 × *g* for 5 min. to remove medium with excess dye, and the pellets were resuspended in PBS and added to a 96-well plate. Fluorescence intensity was measured using a Spectramax M3 plate reader (Molecular Devices, Sunnyvale, CA, USA) at an excitation of 485 nm and emission of 530 nm.

### Mitochondrial ROS levels

To quantify mitochondrial ROS, cells (2** × **10^5^ cells/well) in the 96-well culture plate were incubated with the mitochondrial superoxide–sensitive fluorescent dye MitoSOX Red (1 μM, Invitrogen) for 20 min. at 37**°**C. The intensity of fluorescence was measured using a Spectramax M3 microplate reader (Molecular Devices) at 510 and 580 nm for excitation and emission respectively.

### Statistical analysis

Data are presented as means ± SEM. Statistical significance was evaluated using one-way anova, followed by Tukey's multiple comparison *post hoc* test or an unpaired two-tailed Student's *t*-test. Differences were considered to be statistically significant when *P* *<* 0.05.

## Results

### Metformin attenuates AngII-induced hypertrophy and cell death

In the first set of experiments, we examined whether metformin exerts a direct anti-hypertrophic effect on AngII-treated cardiomyocytes. As previous studies demonstrate that metformin at high concentrations (≥5 mM) reduces the energy state of intact hearts 4, but at 2 mM, it exerts no direct effect on complex I activity 13 and high-energy phosphates 14, we used metformin at a concentration of 2 mM. Likewise, this study revealed no detrimental effects of 2 or 5 mM metformin on complex I activity in H9c2 cardiomyocytes (see below).

**Figure 1:**
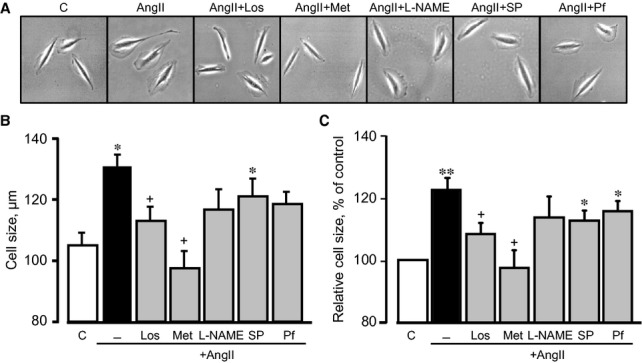
The effects of metformin (Met) on cell size. Cell size was measured in H9c2 cells 24 hrs after treatment with AngII (200 nM) in the presence or absence of losartan (Los), Met, Nω-nitro-L-arginine methyl ester (L-NAME) and splitomicin (SP) or pifithrin-α (Pf). (A) Representative images of individual cells, (B) cell size (μm), (C) cell size presented as percentage of control. *P < 0.05, **P < 0.01 *versus* control; ^+^*P* < 0.05 *versus* AngII. *n* = 3–4 per group.

**Figure 2:**
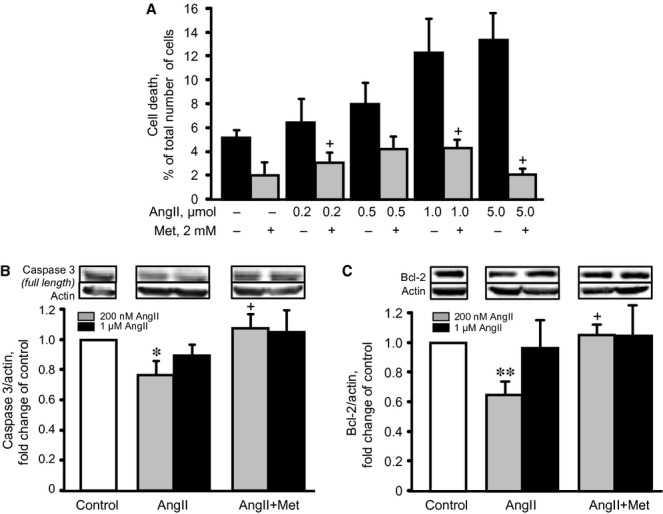
The effects of metformin (Met) on AngII-induced cell death. (A) The effect of AngII (24 hrs), at concentrations ranging from 0 to 5 μM, on cell death, in the presence or absence of 2 mM Met was measured by Trypan blue. (B and C) The effects of Met on caspase 3 (full size) and Bcl-2 protein levels in cells treated with 200 nM or 1 μM AngII. *P < 0.05, **P < 0.01 *versus* control; ^+^*P* < 0.05 *versus* AngII. *n* = 3–4 per group (for apoptotic proteins) and 6–8 (for cell death) per group.

As shown in Figure 1, cells treated for 24 hrs with 200 nM AngII demonstrated significant hypertrophy and a 22% (*P* *<* 0.01) increase in cell size. Both metformin and losartan prevented cell hypertrophy significantly, reducing cell size in the presence of AngII. Cell size values were not significantly affected in cells pre-treated with L-NAME, splitomicin or pifithrin-α. To assess the effect of metformin on cell death, the cells were pre-treated with 2 mM metformin in the presence of AngII at different concentrations (0 nM, 200 nM, 500 nM, 1 μM or 5 μM). AngII-induced cell death was dose-dependent and, when treated with 5 μM AngII, reached a peak rate of 13.3% (Fig. 2A). Metformin prevented cell death at all concentrations of AngII and, interestingly, demonstrated anti-apoptotic effects on cells treated with 200 nM AngII, preventing caspase 3 cleavage and Bcl-2 down-regulation. However, when the cells were treated with 1 μM AngII, the presence or absence of metformin did not alter caspase 3 or Bcl-2 protein levels (Fig. 2B and C), suggesting that cell death occurs through necrosis, rather than apoptosis, at high concentrations of AngII. Thus, these data demonstrate that metformin exerts a direct, anti-hypertrophic effect on cardiomyocyte hypertrophy and prevents AngII-induced cell death.

### Metformin decreases AngII-induced up-regulation of AT1R

**Figure 3:**
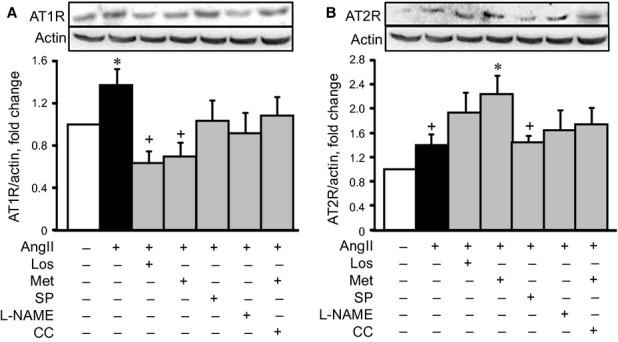
The effect of losartan (Los), metformin (Met), Nω-nitro-L-arginine methyl ester (L-NAME) or splitomicin (SP) on AngII type 1 (AT1R, A) and type 2 (AT2R, B) receptors in cell hypertrophy. Western blot data were normalized to actin and expressed as fold change relative to control groups. *P < 0.05 *versus* control; ^+^*P* < 0.05 *versus* Met. *n* = 6–8 per group.

AT1R is a predominant AngII receptor in cardiac cells that is typically coupled to Gα_q/11_ and activates PKC, Ras, MAPKs, JAK-STAT and other pro-hypertrophy signalling molecules 15. In the first set of experiments, we examined whether metformin affected AT1R levels in AngII-treated H9c2 cells. As shown in Figure 3A, AngII up-regulated AT1R and AT2R by 38% (*P* *<* 0.05) and 40% (*P* *<* 0.05) respectively. The presence of metformin or losartan completely prevented AT1R up-regulation. Also, losartan and metformin further increased AT2R levels by 94% (*P* = *N.S*.) and 124% (*P* *<* 0.05), respectively, in AngII-treated cells, compared with the control group (Fig. 3B). Neither the presence of compound C, splitomicin, nor L-NAME significantly affected AT2R levels. Thus, our data demonstrate that metformin reduces AT1R and increases AT2R in AngII-treated cells. Compound C (AMPK inhibitor) abolished these effects in the presence of metformin, suggesting that anti-hypertrophic effects of metformin require AMPK activation.

### Inhibition of AT1R is associated with AMPK activation

**Figure 4:**
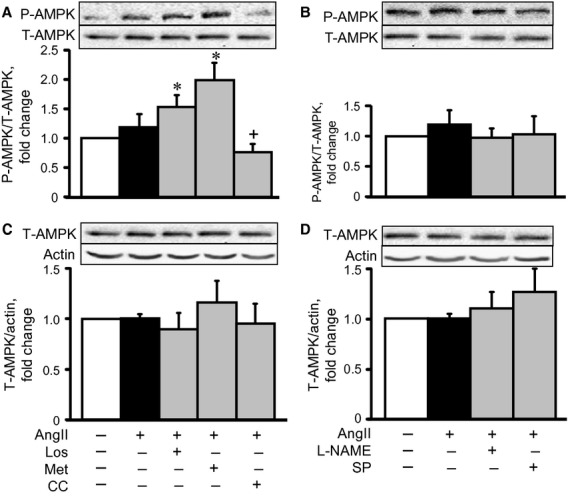
The effects of losartan (Los), metformin (Met), compound C (CC), Nω-nitro-L-arginine methyl ester (L-NAME) or splitomicin (SP) on phospho-AMPK (P-AMPK, A and B) and total-AMPK (T-AMPK, C and D) in cell hypertrophy. Western blot data were normalized to T-AMPK (for P-AMPK) and actin (for T-AMPK) and expressed as fold change relative to control groups. *P < 0.05 *versus* control; ^+^*P* < 0.05 *versus* Los and Met. *n* = 6–8 per group.

In the next group of experiments, we examined whether AT1R inhibition induces AMPK activation in cardiomyocytes. Phosphorylation of AMPK was accepted as an indicator of enzymatic activity, based on previous data showing that phosphorylation at Thr172 correlates with AMPK activation 16. AngII had no significant effect on P-AMPKα^Thr172^, indicating no activation (Fig. 4A). Pre-treatment with losartan and metformin increased AMPK phosphorylation by 54% (*P* *<* 0.05) and 99% (*P* *<* 0.05), respectively, compared with the control group. Neither treatment with splitomicin nor L-NAME affected AMPK phosphorylation (Fig. 4A and B). It should be noted that AngII, in the presence or absence of the pharmacological agents, did not affect total AMPK levels (Fig. 4C and D). Thus, these findings demonstrate that losartan may reduce AngII-induced cardiomyocyte hypertrophy, at least partially, *via* AMPK activation.

### Metformin and losartan increase eNOS activation and reduce p53 in AngII-induced hypertrophy

**Figure 5:**
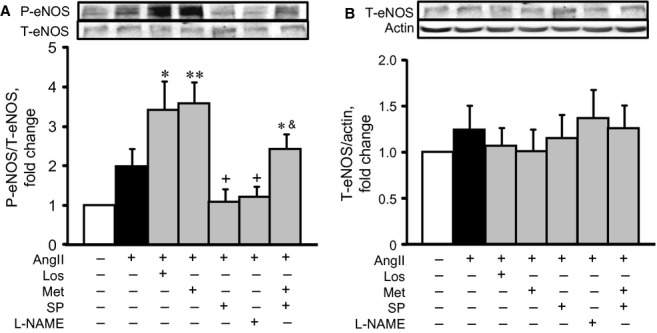
The effects of AngII on phospho-eNOS (P-eNOS, A) and total-eNOS (T-eNOS, B) in cardiomyocytes in the presence or absence of losartan (Los), metformin (Met), Nω-nitro-L-arginine methyl ester (L-NAME), and/or splitomicin (SP). Western blot data were normalized to actin and expressed as fold change relative to control groups. *P < 0.05, **P < 0.01 *versus* control; ^+^*P* < 0.05 *versus* AngII; ^&^*P* < 0.05 *versus* AngII+Met. *n* = 8–12 per group.

Previous studies demonstrate that AMPK phosphorylates eNOS at Ser1177 and increases nitric oxide production in rat hearts 7. Nitric oxide has opposite effects on cell metabolism and mitochondrial function; it protects cells against oxidative stress at low concentrations but exerts detrimental effects at high concentrations 17,18. Recent studies demonstrate that eNOS activation (P-eNOS^Ser1177^) plays a central role in H_2_O_2_- and AngII-induced signalling in cardiac cells 19. In our experiments, incubation of cells with AngII enhanced eNOS phosphorylation by 2.1 fold (*P* *<* 0.05), compared with the control group (Fig. 5A). Surprisingly, both losartan and metformin further increased eNOS activation by 3.3 (*P* *<* 0.05) and 3.6 fold (*P* *<* 0.05) respectively. Pre-treatment with splitomicin or L-NAME completely prevented AngII-induced phosphorylation. AngII did not affect the total level of eNOS in the presence or absence of the pharmacological agents (Fig. 5B).

**Figure 6:**
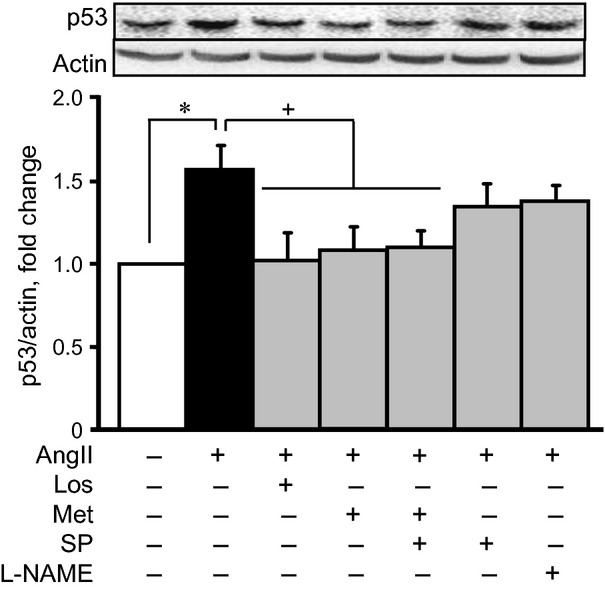
The effects of AngII on the level of p53 in cardiomyocytes in the presence or absence of losartan (Los), metformin (Met), Nω-nitro-L-arginine methyl ester (L-NAME) and/or splitomicin (SP). Western blot data were normalized to actin and expressed as fold change relative to control groups. *P < 0.05 *versus* control; ^+^*P* < 0.05 *versus* AngII. *n* = 10–12 per group.

Recent studies identified p53, a well-known tumour suppressor protein, as a key player in heart failure. It is activated in response to cellular stress, promoting apoptosis and development of heart failure 20. AngII-induced hypertrophy up-regulated p53 and pro-apoptotic signalling pathways in cardiomyocytes 11,21. To the best of our knowledge, there are no studies about the role of AMPK activation in AngII-induced p53 up-regulation. Our studies demonstrated that AngII increased the p53 levels by 58% (*P* *<* 0.05), after 24 hrs of treatment (Fig. 6). Both metformin and losartan prevented p53 up-regulation in AngII-treated cells. SIRT1 regulates p53 activity through acetylation/deacetylation 22,23. Pre-treatment of cells with splitomicin or L-NAME did not significantly change p53 levels (Fig. 6). Thus, these findings demonstrate that metformin reduces AngII-induced hypertrophy in cardiomyocytes through the regulation of the AMPK/SIRT1/eNOS/p53 pathway.

### Metformin prevents mitochondrial membrane depolarization and increases the electron transfer chain (ETC) complex I activity in AngII-treated cardiomyocytes

**Figure 7:**
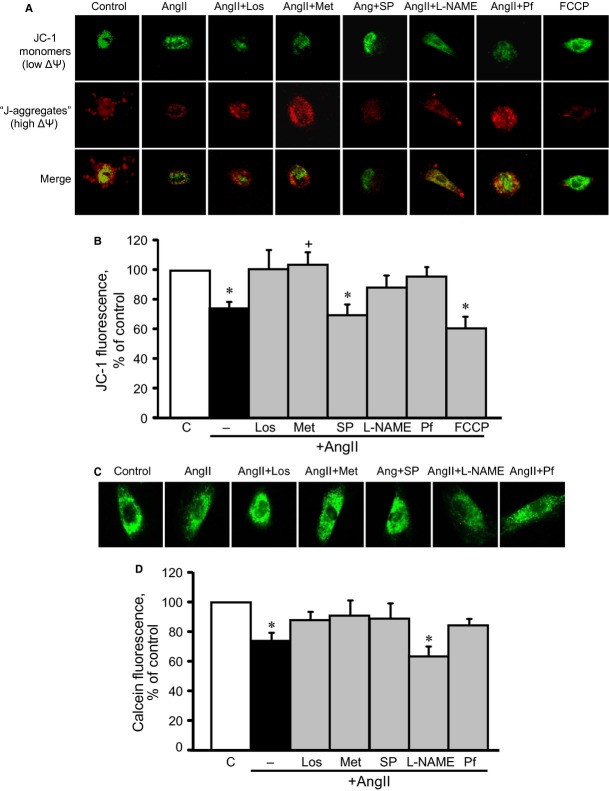
Effects of losartan (Los), metformin (Met), Nω-nitro-L-arginine methyl ester (L-NAME), splitomicin (SP) or pifithrin-α (Pf) on mitochondrial membrane potential (ΔΨ_m_) and permeability transition pores (PTP) in AngII-treated cardiomyocytes. (A) Representative images of JC-1 fluorescence. The images were obtained using a Zeiss LSM510 META (Carl Zeiss) microscope from cells after incubation with JC-1 (10 μg/ml). The cells were treated with Los, Met, SP, L-NAME or Pf in the presence or absence of 200 nM AngII. In addition, to demonstrate specificity of the fluorescence signal, the cells were treated with FCCP (carbonylcyanide-p-trifluoromethoxyphenylhydrazone), a chemical uncoupler of electron transport and oxidative phosphorylation that induces depolarization of the mitochondrial inner membrane. Red fluorescent images of dye aggregates (‘J-aggregates’) indicate high ΔΨ_m_, whereas green images of monomeric dye (‘JC-1 monomers’) show low ΔΨ_m_. (B) Quantitative results of JC-1 fluorescence intensity, measured using a Spectramax M3 microplate reader (Molecular Devices). Data were normalized to cells and expressed as a percentage of the control group. *P < 0.05 *versus* control. *n* = 6–8 per group. (C) Representative images of calcein fluorescence. Cells treated with Los, Met, SP, L-NAME or Pf in the presence or absence of 200 nM AngII for 24 hrs were co-loaded with cobalt chloride (5 mM) and calcein-AM (5 μM) and, then, imaged using an Olympus IX73 (Center Valley, PA, USA) inverted fluorescence microscope. (D) Quantitative results of calcein fluorescence, normalized to individual cells and expressed as a percentage of the control group. *P < 0.05 *versus* control; *n* = 4 per group.

**Figure 8:**
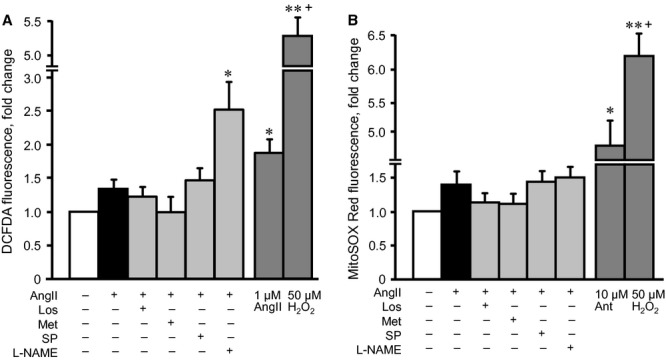
Effects of losartan (Los), metformin (Met), Nω-nitro-L-arginine methyl ester (L-NAME) or splitomicin (SP) on total and mitochondrial reactive oxygen species (ROS) levels in AngII-treated H9c2 cells. (A) Total ROS levels were determined in the cells incubated with the ROS-specific probe, DCF-DA, after treatment with 200 nM AngII in the presence or absence of Los, Met, L-NAME or SP. In additional groups, the cells were treated with 50 μM H_2_O_2_ (positive control) or 1 μM AngII for 24 hrs. (B) Mitochondrial ROS levels were measured under the same conditions using the mitochondrial-sensitive fluorescence dye, MitoSOX Red. In additional groups, the cells were treated with 50 μM H_2_O_2_ or 10 μM antimycin (positive control) for 24 hrs. Results were normalized per 10^6^ cells (for total ROS) and 10^3^ cells (for mitochondrial ROS) and expressed as a percentage of the control group. *P < 0.05 *versus* control; *n* = 6–8 per group.

**Figure 9:**
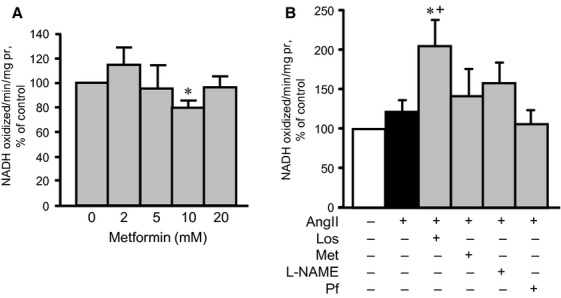
Effects of losartan (Los), metformin (Met), Nω-nitro-L-arginine methyl ester (L-NAME) or splitomicin (SP) on the activity of ETC. complex I, in the presence or absence of AngII. (A) Met, at concentrations ranging from 0 to 20 mM, was added directly to cell lysates isolated from control H9c2 cardiomyocytes, and the activity of complex I was measured. (B) The complex I activity was measured in cardiomyocytes treated with 200 nM AngII, in the presence or absence of Los, Met, L-NAME or SP. Complex I activity was measured by monitoring the decrease in NADH concentration, normalized to mg of protein and expressed as a percentage of the control group.

Hypertrophic signalling pathways mostly converge on mitochondria to regulate energy metabolism in response to various cellular stresses. AMPK plays a central role in mitochondrial metabolism and ATP production by stimulating glucose uptake, fatty acid oxidation and glycolysis. We examined whether metformin prevents AngII-induced mitochondrial dysfunction in H9c2 cells. As shown in Figure 7A and B, AngII reduced the membrane potential of mitochondria by 26% (*P* *<* 0.05). All drugs, except splitomicin, prevented AngII-induced membrane depolarization. Particularly, pifithrin-α prevented membrane depolarization, indicating that AngII-induced mitochondrial dysfunction may be associated with p53 activation. Indeed, p53 translocation to mitochondria may be associated with mitochondrial dysfunction and cell death, according to numerous studies performed by various groups 24,25. Furthermore, physical interaction between p53 and cyclophilin D (CyP-D), a regulatory protein of the mitochondrial PTP, was found in the mitochondria of cancer cells 24 and in brains subjected to ischaemia/reperfusion 25. However, our experiments with immunoprecipitation of p53 followed by immunoblotting of CyP-D revealed no physical interaction between these two proteins or other possible PTP components, such as adenine nucleotide translocase and voltage-dependent anion channels (data not shown). In addition, metformin and other pharmacological agents, except L-NAME, prevented AngII-induced PTP opening (Fig. 7C and D). Interestingly, however, AngII did not induce a significant increase in either total or mitochondrial ROS production in the presence or absence of the tested pharmacological agents, except L-NAME, which significantly increased total ROS levels (Fig. 8A and B). Metformin may mediate its beneficial effects through direct inhibition of the ETC complex I activity. To elucidate this hypothesis, we assessed a direct effect of metformin on complex I activity in untreated cells (not pre-treated with AngII or pharmacological agents), at concentrations ranging from 0 to 20 mM. Low concentrations (2 and 5 mM) of metformin did not affect complex I activity, but 10 mM metformin inhibited it (Fig. 9A). Interestingly, AngII alone did not significantly change complex I activity. However, pre-treatment of AngII-treated cells with metformin increased the activity of the complex (Fig. 9B). Thus, metformin prevents depolarization of mitochondria, inhibits PTP opening and improves ETC activity in AngII-treated cells. The beneficial effects of metformin on mitochondria are not associated with its direct interaction with complex I of the ETC.

## Discussion

**Figure 10:**
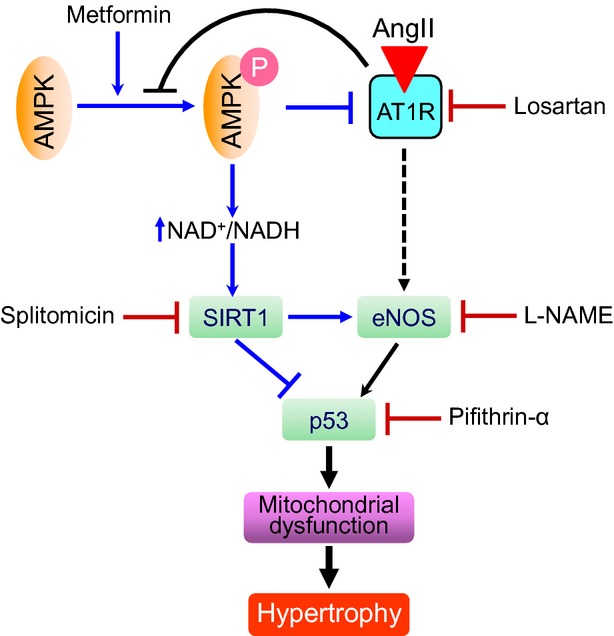
Proposed mechanism of the anti-hypertrophic effect of metformin on AngII-induced hypertrophy.

The results of this study demonstrate the following: (*i*) In response to AngII, there is a negative reciprocal relationship between AMPK and AT1R. Metformin activates AMPK, inducing down-regulation of AT1R, whereas losartan blocks AT1R and stimulates AMPK activation. (*ii*) Both AMPK activation and AT1R inhibition increase eNOS phosphorylation and down-regulate p53. (*iii*) The beneficial effects of AMPK activation and AT1R inhibition may be associated with the prevention of mitochondrial dysfunction in cardiomyocytes. In Figure 10, we propose a mechanism for the anti-hypertrophic effect of metformin on AngII-induced cardiomyocyte hypertrophy. Previous studies demonstrating anti-hypertrophic and anti-remodelling effects of metformin were mostly performed *in vivo* on intact hearts 7,26. However, along with cardiomyocytes, intact hearts contain other cell types, including endothelial and smooth muscle cells, fibroblasts and other connective tissue cells, mast cells and immune system–related cells. This study attempts to clarify the role of cardiomyocytes in the anti-hypertrophic action of metformin.

Metformin is a well-known AMPK activator, and accumulated data demonstrate that AMPK activation plays a central role not only in cardioprotection against ischaemia/reperfusion injury 27 but also in induction of anti-hypertrophic/anti-remodelling effects 28. Currently, there are no studies elucidating the relationship between AMPK and AT1R in cardiomyocyte hypertrophy. Our data demonstrate that metformin reduced the amount of AT1R in cultured cardiomyocytes; thus, it blocked the hypertrophic effects of AngII. The effect of metformin was attributed to AMPK activation, as the presence of compound C down-regulated AT1R. AMPK may reduce AT1R levels through down-regulation of genes encoding the receptor, followed by P-AMPK translocation to the nucleus. In response to stress, AMPK subunits accumulate in nuclei, independent of kinase activation 29, where the nuclear AMPKα2 complex regulates gene transcription 30. In addition, active AMPK may abrogate the effect of AngII by inhibiting NAD(P)H oxidase, a downstream target of AT1R, as shown in endothelial cells 31. In our experiments, losartan stimulated AMPK phosphorylation, suggesting that a negative reciprocal relationship may exist between AMPK and AT1R. It should be noted that beneficial effects of metformin may also be mediated through AT2R stimulation, which would counteract AT1R and abrogate AT1R-mediated growth responses in cardiac hypertrophy 32. Previous studies demonstrate an increase 33, decrease 34 or no change 35 in AngII-induced activation of AMPK in cardiac cells. We observed that AngII did not remarkably affect AMPK phosphorylation; however, losartan significantly stimulated AMPK phosphorylation by a yet unknown mechanism. Our data are consistent with previous studies, which show that administration of the AT1R blockers, telmisartan and candesartan stimulated AMPK activity in cultured myotubes 36 and rat hypothalamuses 37. It remains unclear how AT1R inhibition stimulates AMPK. We are tempted to speculate that AT1R-induced AMPK activation may be mediated through regulation of the upstream molecules, LKB1 and/or CamKK, and internalization and interaction of AT1R with AMPK.

The beneficial effects of AMPK activation and AT1R inhibition on mitochondria may be mediated through the same mechanism(s) or independent pathways. AngII binds to AT1R, activating the transcription factor, p53, and apoptosis. p53 binding to AT1R gene promoter regions is primarily responsible for up-regulating the receptor 38. We found that losartan prevention of AT1R up-regulation was associated with decreased p53 levels, indicating that the latter mediates the AT1R-induced apoptotic pathway. On the other hand, metformin- or losartan-induced AMPK activation can increase NAD^+^/NADH, which, in turn, stimulates the NAD^+^-dependent deacetylases sirtuins, including SIRT1, a regulator of p53 activity. According to previous studies, when metformin induces activation of AMPK and SIRT1, an inverse relationship exists between metformin-triggered AMPK-SIRT1 signalling and p53 protein levels in high glucose-exposed HepG2 cells 39. Accordingly, in the presence of metformin, AMPK-induced p53 down-regulation may prevent AngII-induced AT1R up-regulation 38.

Phosphorylation of eNOS at Ser1177 may be a downstream target of the AMPK/SIRT1 pathway 40. Remarkably, both metformin and losartan increased P-eNOS^Ser1177^ levels in our studies. Splitomicin, however, prevented eNOS activation, indicating that SIRT1 is an upstream molecule of eNOS. Although eNOS inhibition enhanced AMPK activation in endothelial cells 41, our data revealed that treatment with L-NAME did not appreciably alter P-AMPK levels in cardiomyocytes. This difference can be due to cell specificity, as different cell types were used in these studies. Activation of the AMPK/eNOS pathway and subsequent metformin- or losartan-enhanced nitric oxide production may have various, further cardioprotective effects. In addition to coronary vasodilatation, nitric oxide regulates cell metabolism, including mitochondrial function in cardiomyocytes 42.

A number of studies 6,43, including ours, 13 demonstrate that metformin therapy attenuates mitochondrial dysfunction during ischaemia/reperfusion and heart failure *via* yet unknown mechanisms. Consistent with these reports, we found that metformin prevented AngII-induced depolarization of the mitochondrial membrane, an index of mitochondrial function in cardiomyocytes. Likewise, both losartan and pifithrin-α may improve mitochondrial function, indicating that the effects of AMPK activation and AT1R inhibition converge on mitochondria. Various mechanisms can mediate the beneficial effects of AMPK activation on mitochondria. For example, metformin protects the heart through PI3K- 43 and PPARα- 13 mediated inhibition of the mitochondrial PTP. On the other hand, in response to oxidative stress, p53 translocates to mitochondria and forms the CyP-D-p53 complex, which is associated with mitochondrial PTP opening 24,25. In the presence or absence of metformin, we found no difference between control and AngII-treated groups with regard to the interaction of CyP-D with p53, although pifithrin-α abrogated AngII-induced membrane depolarization. These results indicate that metformin-induced activation of the AMPK/SIRT1/eNOS pathway prevents AngII-induced, p53-dependent membrane depolarization and PTP opening in mitochondria, which is not associated with protein–protein interaction between p53 and CyP-D. It appears that further studies are necessary to clarify the role of p53 in hypertrophy-induced mitochondrial dysfunction. At high concentrations (>10 mM), metformin may improve mitochondrial function, independent of AMPK activation *via* direct inhibition of complex I 44. However, this study excluded that possibility, because 2 mM metformin did not inhibit complex I activity (Fig. 9A).

In conclusion, metformin activates AMPK, preventing AT1R up-regulation, and, *vice versa,* losartan down-regulates AT1R, stimulating AMPK. As such, this study demonstrates a negative reciprocal relationship between AMPK activation and AT1R levels in AngII-induced cardiomyocyte hypertrophy. In response to AngII administration, the AMPK/SIRT1/eNOS pathway abrogation of mitochondrial dysfunction mediates, at least partially, the beneficial effects of metformin and losartan. Our studies provide further evidence that AMPK can be targeted to promote cardiomyocyte survival *via* activation of several signalling pathways in hypertrophy and heart failure.
